# Supplementary material for: From Stoop to Squat: A Comprehensive Analysis of Lumbar Loading Among Different Lifting Styles
Source: Front Bioeng Biotechnol. 2021 Nov 4;9:769117. doi: 10.3389/fbioe.2021.769117 (PMC8599159; doi:10.3389/fbioe.2021.769117)
Supplement: Supplementary file 1 [file DataSheet1.PDF]

Supplementary Table 1: Results from the statistical analysis of continuous loads. Presented are F-Threshold (F), number of clusters (n) and cluster location (location) throughout the lift cycle and p-values for each cluster from the post hoc analysis with SPM. Non-significant results are not shown and left empty (-)

LOAD	FREESTYLE VS SQUAT				FREESTYLE VS STOOP				SQUAT VS STOOP			
**TOTAL**	**F**	**n**	**Location**	**p-Values**	**F**	**n**	**Location**	**p-Values**	**F**	**n**	**Location**	**p-Values**
T12_L1	-	-	-	-	3.76	2	0-64%<br>98-100%	p=0<br>p=0.015	3.82	1	0-66%	p=0
L1_L2	-	-	-	-	3.78	2	0-59%<br>98-100%	p=0<br>p=0.014	3.81	1	0-57%	p=0
L2_L3	-	-	-	-	3.80	2	0-54%<br>97-100%	p=0<br>p=0.013	3.81	1	0-47%	p=0
L3_L4	-	-	-	-	3.82	2	0-48%<br>97-100%	p=0<br>p=0.013	3.79	1	0-38%	p=0
L4_L5	3.61	3	0-3%<br>5-9%<br>16-35%	p=0.016<br>p=0.014<br>p<0.001	3.83	2	0-49%<br>98-100%	p=0<br>p=0.014	3.79	1	1-33%	p<0.001
L5_S1	3.62	2	0-12%<br>14-35%	p=0.003<br>p<0.001	3.84	2	0-50%<br>98-100%	p=0<br>p=0.014	3.79	1	1-33%	p<0.001
**COMPRESSIVE**												
T12_L1	-	-	-	-	3.77	2	0-65%<br>98-100%	p=0<br>p=0.015	3.83	1	0-66%	p=0
L1_L2	-	-	-	-	3.79	2	0-60%<br>98-100%	p=0<br>p=0.014	3.82	1	0-59%	p=0
L2_L3	-	-	-	-	3.80	2	0-55%<br>97-100%	p=0<br>p=0.014	3.81	1	0-49%	p=0
L3_L4	-	-	-	-	3.81	2	0-50%<br>97-100%	p=0<br>p=0.013	3.79	1	0-40%	p=0
L4_L5	3.61	3	0-2%<br>6.9-7%<br>17-34%	p=0.016<br>p=0.017<br>p<0.001	3.83	2	0-50%<br>98-100%	p=0<br>p=0.014	3.79	1	0-34%	p=0
L5_S1	3.62	3	0-2%<br>7-8%<br>17-40%	p=0.016<br>p=0.017<br>p<0.001	3.85	2	0-46%<br>98-100%	p=0<br>p=0.014	3.80	1	1-29%	p<0.001

Supplementary Table 1: Cont.

LOAD	FREESTYLE VS SQUAT				FREESTYLE VS STOOP				SQUAT VS STOOP			
**AP SHEAR**												
T12_L1	*3.50*	*1*	*0-91%*	*p=0*	*3.63*	*1*	*0-92%*	*p=0*	*3.65*	*2*	*0-95%*<br>*99-100%*	*p=0*<br>*p=0.017*
L1_L2	*3.48*	*1*	*0-92%*	*p=0*	*3.63*	*1*	*0-90%*	*p=0*	*3.65*	*1*	*0-94%*	*p=0*
L2_L3	*3.44*	*1*	*0-93%*	*p=0*	*3.46*	*1*	*0-91%*	*p=0*	*3.47*	*1*	*0-95%*	*p=0*
L3_L4	*3.46*	*1*	*0-86%*	*p=0*	*3.52*	*1*	*0-86%*	*p=0*	*3.54*	*1*	*0-90%*	*p=0*
L4_L5	*3.56*	*1*	*5-79%*	*p=0*	*3.69*	*3*	*0-3%*<br>*25-67%*<br>*97-100%*	*p=0.014*<br>*p<0.001*<br>*p=0.015*	*3.64*	*2*	*0-79%*<br>*97-100%*	*p=0*<br>*p=0.015*
L5_S1	*3.62*	*1*	*0-30%*	*p<0.001*	*3.75*	*2*	*0-64%*<br>*98-100%*	*p=0*<br>*p=0.015*	*3.68*	*2*	*1-50%*<br>*98-100%*	*p=0*<br>*p=0.016*

Supplementary Table 2: Results from the statistical analysis of peak loads. Displays descriptive values (mean, sd) of discrete outcome variables for peak loads (total, compressive and AP shear) per segment and corresponding p-values from ANOVA and post hoc tests. Significance is indicated by *.

LOAD	FREESTYLE *mean (sd)*	SQUAT *mean (sd)*	STOOP *mean (sd)*	ANOVA	FREESTYLE VS SQUAT	FREESTYLE VS STOOP	SQUAT VS STOOP
**TOTAL**							
T12_L1	*4.23 (0.89)*	*4.25 (0.50)*	*3.08 (0.40)*	*p<0.001**	*p=0.325*	*p<0.001**	*p<0.001**
L1_L2	*4.43 (0.77)*	*4.31 (0.44)*	*3.42 (0.38)*	*p<0.001**	*p=0.513*	*p<0.001**	*p<0.001**
L2_L3	*4.58 (0.64)*	*4.38 (0.41)*	*3.76 (0.38)*	*p<0.001**	*p=0.062*	*p<0.001**	*p<0.001**
L3_L4	*4.77 (0.60)*	*4.49 (0.43)*	*4.04 (0.42)*	*p<0.001**	*p=0.002**	*p<0.001**	*p<0.001**
L4_L5	5.64 (0.63)	5.26 (0.48)	4.88 (0.49)	*p<0.001**	*p<0.001**	*p<0.001**	*p<0.001**
L5_S1	5.95 (0.61)	5.55 (0.48)	5.15 (0.53)	*p<0.001**	*p<0.001**	*p<0.001**	*p<0.001**
**COMPRESSIVE**							
T12_L1	*4.20 (0.90)*	*4.24 (0.50)*	*3.02 (0.39)*	*p<0.001**	*p=0.238*	*p<0.001**	*p<0.001**
L1_L2	*4.42 (0.78)*	*4.30 (0.44)*	*3.38 (0.39)*	*p<0.001**	*p=0.636*	*p<0.001**	*p<0.001**
L2_L3	*4.57 (0.65)*	*4.37 (0.41)*	*3.70 (0.38)*	*p<0.001**	*p=0.084*	*p<0.001**	*p<0.001**
L3_L4	*4.73 (0.61)*	*4.47 (0.43)*	*3.98 (0.42)*	*p<0.001**	*p=0.004**	*p<0.001**	*p<0.001**
L4_L5	5.56 (0.63)	5.19 (0.48)	4.78 (0.49)	*p<0.001**	*p<0.001**	*p<0.001**	*p<0.001**
L5_S1	5.35 (0.61)	5.00 (0.51)	4.69 (0.54)	*p<0.001**	*p<0.001**	*p<0.001**	*p<0.001**
**AP SHEAR**							
T12_L1	*-0.40 (0.37)*	*0.15 (0.30)*	*-0.87 (0.15)*	*p<0.001**	*p<0.001**	*p<0.001**	*p<0.001**
L1_L2	*-0.32 (0.30)*	*-0.09 (0.28)*	*-0.68 (0.14)*	*p<0.001**	*p<0.001**	*p<0.001**	*p<0.001**
L2_L3	*-0.15 (0.35*	*0.09 (0.26)*	*-0.71 (0.19)*	*p<0.001**	*p<0.001**	*p<0.001**	*p<0.001**
L3_L4	*-0.54 (0.22)*	*-0.39 (0.18)*	*-0.78 (0.12)*	*p<0.001**	*p<0.001**	*p<0.001**	*p<0.001**
L4_L5	-0.93 (0.26)	-0.85 (0.26)	-1.01 (0.21)	*p<0.001**	*p<0.001**	*p<0.001**	*p<0.001**
L5_S1	-2.51 (0.48)	-2.35 (0.44)	-2.09 (0.48)	*p<0.001**	*p<0.001**	*p<0.001**	*p<0.001**

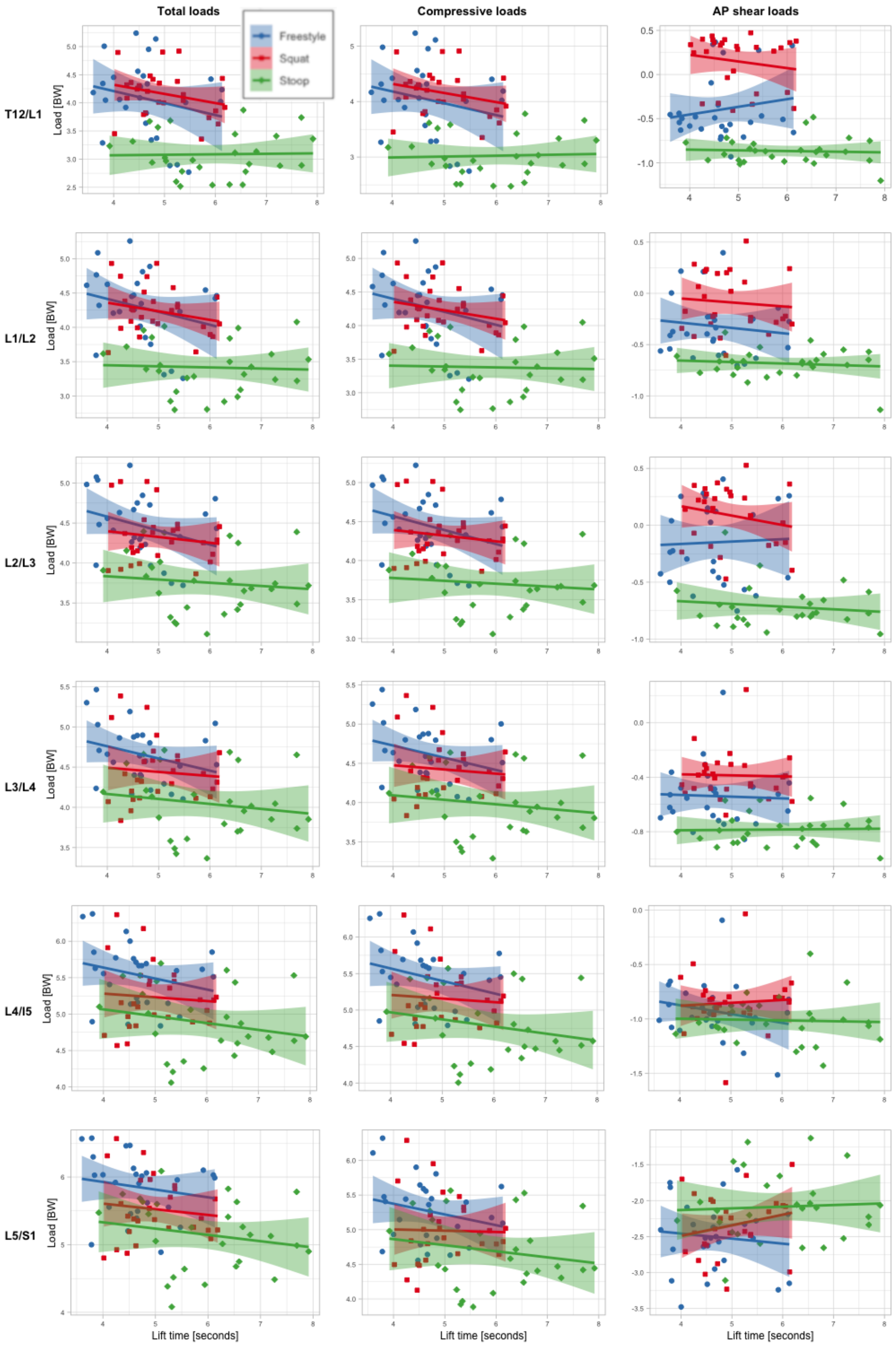

Supplementary Figure 1. Scatterplots display specific peak loads in relation to lift time. Lifting style loads are grouped by color. Colored lines indicate regression lines for respective styles, standard error is indicated by the shaded band around the regression line.
